# Screening Microbial Interactions During Inulin Utilization Reveals Strong Competition and Proteomic Changes in *Lacticaseibacillus paracasei* M38

**DOI:** 10.1007/s12602-023-10083-5

**Published:** 2023-05-25

**Authors:** Marco Vega-Sagardía, Eva Cebrián Cabezón, Josué Delgado, Santiago Ruiz-Moyano, Daniel Garrido

**Affiliations:** 1 Department of Chemical and Bioprocess Engineering, School of Engineering, Pontificia Universidad Católica de Chile, Vicuña Mackenna 4860, Santiago, Chile; 2 Facultad de Veterinaria, Higiene y Seguridad Alimentaria, Instituto Universitario de Investigación de Carne y Productos Cárnicos, Universidad de Extremadura, Avda. de las Ciencias s/n, 10003 Cáceres, Spain; 3 Departamento de Producción Animal y Ciencia de los Alimentos, Nutrición y Bromatología, Escuela de Ingenierías Agrarias, Universidad de Extremadura, Avda. Adolfo Suárez s/n, 06007 Badajoz, Spain; 4 Instituto Universitario de Investigación de Recursos Agrarios (INURA), Universidad de Extremadura, Avda. de la Investigación s/n, Campus Universitario, 06006 Badajoz, Spain

**Keywords:** *Bifidobacterium*, *Bacteroides*, *Lactobacillus*, Inulin, Prebiotic, Proteomics

## Abstract

Competition for resources is a common microbial interaction in the gut microbiome. Inulin is a well-studied prebiotic dietary fiber that profoundly shapes gut microbiome composition. Several community members and some probiotics, such as *Lacticaseibacillus paracasei*, deploy multiple molecular strategies to access fructans. In this work, we screened bacterial interactions during inulin utilization in representative gut microbes. Unidirectional and bidirectional assays were used to evaluate the effects of microbial interactions and global proteomic changes on inulin utilization. Unidirectional assays showed the total or partial consumption of inulin by many gut microbes. Partial consumption was associated with cross-feeding of fructose or short oligosaccharides. However, bidirectional assays showed strong competition from *L. paracasei* M38 against other gut microbes, reducing the growth and quantity of proteins found in the latter. *L. paracasei* dominated and outcompeted other inulin utilizers, such as *Ligilactobacillus ruminis* PT16, *Bifidobacterium longum* PT4, and *Bacteroides fragilis* HM714. The importance of strain-specific characteristics of *L. paracasei*, such as its high fitness for inulin consumption, allows it to be favored for bacterial competence. Proteomic studies indicated an increase in inulin-degrading enzymes in co-cultures, such as β-fructosidase, 6-phosphofructokinase, the PTS D-fructose system, and ABC transporters. These results reveal that intestinal metabolic interactions are strain-dependent and might result in cross-feeding or competition depending on total or partial consumption of inulin. Partial degradation of inulin by certain bacteria favors coexistence. However, when *L. paracasei* M38 totally degrades the fiber, this does not happen. The synergy of this prebiotic with *L. paracasei* M38 could determine the predominance in the host as a potential probiotic.

**Supplementary Information:**

The online version contains supplementary material available at 10.1007/s12602-023-10083-5.

## Introduction

The gut microbiome comprises the collective genome of microbes that inhabit the gut, including bacteria, archaea, viruses, and fungi [1]. These microorganisms can provide nutrients and vitamins to the host and protect against colonization by pathogenic microorganisms [2]. The dietary intake of specific non-digestible carbohydrates is increasingly seen as a highly effective approach to manipulating the composition and activities of the human gut microbiota to benefit health [3]. Dietary fibers are complex carbohydrate polymers found in fruits, vegetables, legumes, seeds, and cereals, which endogenous human enzymes cannot hydrolyze. However, the intestinal microbiome can selectively metabolize them through anaerobic fermentation [4, 5].

Some dietary fibers may act as prebiotics, enhancing the proliferation of beneficial microbes in the gut and host health [6]. Its consumption is associated with antidiabetic and antihypertensive properties [7]. The most common prebiotics contained in foods are fructooligosaccharides (FOS) and inulin [6]. Inulin is a fructan polysaccharide (polymer of fructose chains) linked by β-2,1 bonds (between 2 and 60 units) with glucose at its end [8, 9]. Inulin stimulates the growth of bifidobacteria and lactobacilli, which are beneficial for health [10]. It is found in roots and vegetables such as onions, artichokes, and chicory and can be fermented by several bacterial genera, such as *Lactobacillus* [11], *Bifidobacterium* [12, 13], and *Bacteroides* [14]. Multiple studies have shown the benefits of inulin consumption in stimulating the growth of health-promoting species that produce short-chain fatty acids (SCFAs) [15]. Inulin promotes increased intestinal calcium absorption, colonic pH regulation, gastrointestinal transit [16], improves blood lipid profiles, relieving constipation [17, 18] and protecting intestinal barrier function by restoring the microbiome [15].

Enzymes that metabolize inulin belong to the GH32 and GH91 glycosyl hydrolase families. These families include enzymes, such as inulinase, invertase, and levanase. Inulinases act precisely on the β-2,1-linkages of inulin, producing fructose and FOS [19]. These enzymes are classified as exo and endoinulinases [20]. Exoinulinase (fructan β-fructosidase) degrades inulin from its non-reducing terminal end to release the fructose units. *Lacticaseibacillus paracasei* has an inulin gene cluster that includes fructose-PTS system proteins (FosA, FosB, FosC, and FosD) and an extracellular β-fructosidase (FosE) [21]. In contrast, endoinulinase disrupts the internal bonds of inulin to produce smaller FOS [22, 23]. Intracellular β-fructofuranosidases capable of fermenting inulin have been found in *L. paracasei* BGP1 [24], *Bifidobacterium adolescentis* [19], and *Bifidobacterium longum* [25]. When inulin is degraded into FOS, it is transported by the ATP-binding cassette (ABC transport) identified in *B. longum* NCC2705. The fructan metabolic pathway derives fructose from the bifid shunt pathway in these bacteria [26]. This pathway converts these monosaccharides into fructose-6-phosphate, intermediates of the hexose fermentation pathway. In *Lactobacillus*, the pathway proposed by Buntin et al., (2017) [27] for inulin metabolization includes the degradation by β-fructosidase and the entry of FOS (by ABC transporter) or fructose (by PTS transporter) to the cell. Fructose is then catabolized by 1-phosphofructokinase and 6-phosphofructokinase to synthesize β-D-fructose-1,6-bisphosphate. The latter can also be synthesized from FOS by sucrose-6-phosphate hydrolase, fructokinase, and 1-phosphofructokinase. Fructose-bisphosphate aldolase class II converts β-D-fructose-1,6-bisphosphate to glyceraldehyde-3-phosphate, a compound metabolized in glycolysis.

Bacterial interactions are fundamental in shaping the gut microbiome. They can occur between microorganisms of the same species or between different species, genera, families, and domains [1, 28]. Interactions can be positive (cooperation) or negative (competition) [29]. In this context, microbial life strategies can influence the outcomes of interactions [30] and determine various consequences for microbial fitness, population dynamics, and functional capabilities within the microbiome [31]. However, competition is estimated to be prevalent among many bacterial species, but few cooperative interactions [32, 33]. Competition can be passive or active. During passive competition, strain can negatively affect the other through resource competition. In active competition (for resources or space), strains inhibit and kill each other through direct interference [34], bacteriocins, or the production of toxic waste products [35]. Species that compete for similar resources produce antimicrobial compounds for adaptive advantages [36]. For example, *Escherichia coli* K-12, *Lactobacillus johnsonii* NCC533, and *B. longum* NCC2705 [37] in the gut of gnotobiotic mice showed that the addition of *E. coli* Nissle 1917 led to the elimination of *L. johnsonii* and *E. coli* K-12, whereas *B. longum* only decreased its population. On the other hand, *Bifidobacterium animalis* BB04 produces the bacteriocin bifidocin A and acts against *E. coli*, *Listeria monocytogenes*, and *Staphylococcus aureus* [38].

Competition is a phenomenon that can help to understand how bacteria adapt to adverse conditions. The combined synergistic effect of inulin and potential probiotics needs further investigation to understand the impact of dietary fiber on gut bacteria. It is essential to unveil bacteria-bacteria interactions [3, 39]. In this study, we used proteomics on inulin bidirectional cultures to determine the resource competence interactions between *L. paracasei* M38 and other bacteria of different phyla (*Ligilactobacillus ruminis* PT16, *B. longum* PT4, and *Bacteroides fragilis* HM714). Proteomics is a robust platform with great potential for studying antagonistic mechanisms between bacteria, such as pathogen inhibition by *Lactobacillus* [40]. Therefore, studying gut microbiome commensal bacteria used in this investigation could reveal different affinities for inulin metabolism. This knowledge could contribute to a better understanding of the competitive mechanisms of paired bacterial interactions at the gut level in the presence of this dietary fiber.

## Materials and Methods

### Strains and Culture Medium

Among the 16 strains used in this work (Supplementary file Table S1) were *B. fragilis* HM714 (Bf, BEI Resources) and strains isolated from fecal samples of Chilean young adults such as *L. paracasei* M38 (Lp), *L. ruminis* PT16 (Lr), *B. longum* PT4 (Bl1), *B. longum* PT8 (Bl2), and *B. longum* PT33 (Bl3) [41]. The base culture medium used in this work was modified ZMB (mZMB) [42], which is a complex medium of known composition (minerals, ions, and vitamins) and is adequate for the growth of anaerobic bacteria. All the substrates used in modified mZMB, such as L-cysteine (cys; Sigma-Aldrich, St. Louis, MO, USA), inulin (Piping Rock, Ronkonkoma, NY, USA), or lactose (Sigma-Aldrich), were sterilized by filtration through 0.22 µm filters (Jet Biofil, China). Clostridium-reinforced medium (RCM; Becton, Dickinson, Franklin Lakes, NJ, USA) and Man-Rogosa-Sharpe (MRS; Difco Laboratories, Detroit, MI, USA) were autoclaved for 15 min at 121 °C. Bacterial growths in an anaerobic jar (Anaerocult; Merck, Darmstadt, Germany) with anaerobic packs (Gaspak EM; Becton, Dickinson, Franklin Lakes, NJ, USA) were reactivated from a -80 °C stock in a 1 mL (inoculum 8% v/v) of its respective culture medium (Supplementary file Table S1), for 48 h at 37 °C. Next, cultures were centrifuged (5000 × *g*), washed with pre-reduced mZMB medium, and the pellets were dissolved in mZMB medium supplemented with 1% (w/v) inulin (mZMB IN) or 2% (w/v) lactose (mZMB LAC) as a positive control, as appropriate.

### Genomic Search of Inulin-Degrading Enzymes

Microbial strains were sequenced by MicrobesNG (Birmingham, UK) using Illumina MiSeq. *B. longum* PT4 (JAPJDT000000000) and *B. longum* PT8 (JARCPQ000000000) genome sequences were deposited at NCBI. *L. paracasei* M38 genome (PRJNA861286) was previously deposited by Torres-Miranda et al. (2022) [21]. *B. fragilis* HM714 genome was obtained from the Integrated Microbial Genome Database (IMG) [43]. Finally, we individually searched for enzymes of interest related to inulin degradation by Uniprot or literature [27, 44, 45]. Specifically, we searched for enzymes linked to the catabolism of inulin, FOS, and fructose and their transports (ABC and PTS transporters). These include β-fructofuranosidase and 6-phosphofructokinase, enzymes involved in the degradation of fructans.

### Monoculture Growths

Each strain was reactivated and inoculated (8% v/v) following the methodology described by Hirmas et al. (2022) [46], with some modifications. Assays were performed in triplicate to evaluate the consumption of mZMB IN or mZMB LAC. Growth kinetics were performed in 96-well plates covered with a mineral oil layer, and the strains were cultured for 48 h at 37 °C in an anaerobic chamber (Sheldon Manufacturing INC, Bactronez-2 Anaerobic Chamber Workstation, Cornelius, OR, USA). The optical density (OD) at 600 nm was measured in a Tecan F50 spectrophotometer (Tecan Trading AG, Infinite F50, Männedorf, Switzerland) every 30 min with shaking every 5 s before measuring.

### Unidirectional Growths

Unidirectional cultures (using a bacterial supernatant of 24 h for culturing another bacterium) were performed as described above with some modifications. Primary degrader strains (Lp, Bl1, Bl2, Bl3, and Bf) were cultured in mZMB IN under anaerobic conditions for 24 h at 37 °C in 5 mL tubes (8% v/v). The culture was then centrifuged at 10,000 × *g* for 1 min, and the supernatant was sterilized using a 0.22 μm filter. Each bacterium (secondary degrader) grew in the supernatant of the primary degrader for 48 h, as previously indicated. Growths were performed in triplicates and represented as ΔOD = OD_final_ – OD_initial._

### Bidirectional Growths

Bidirectional assays correspond to two bacteria grown simultaneously and separated by a membrane. The strains selected for this assay were those that best degraded inulin in monoculture. The considered pairs were (insert/well): Lr/Lp; Bl1/Lp; Bf/Lp. Both monocultures and bicultures were analyzed. The bacterium with the best growth on inulin (Lp) was plated in the bottom well in 1 mL of mZMB IN. The other bacteria of the evaluated pair (Lr, Bl1, Bf) were grown in the upper insert, which contained 250 μL of mZMB IN. The procedure was performed as described by Hirmas et al. (2022) [46]. Briefly, bacteria were first reactivated in RCM (Bf) or MRS (Lp, Lr, and Bl1) for 48 h, and centrifuged at 10,000 × *g* for 1 min, then washed with the mZMB (without carbon source). Bacteria were then inoculated at 8% v/v in mZMB IN or mZMB LAC (basal treatment), as appropriate, onto Transwell plates (JetBiofil, China). The plates were incubated in an anaerobic jar using anaerobic packs at 37 °C for 48 h. At the end of the experiment, the contents of the Transwell plates were transferred into a 96-well plate, and OD 600 (at 0 h and 48 h) was measured using a Tecan Infinite M200 Pro plate reader (Tecan Trading AG, Grödig, Austria). Finally, the culture was centrifuged at 10,000 × *g* for 1 min, and the supernatant and precipitate were stored at − 80 °C until further use.

### Carbohydrate Consumption

Thin-layer chromatography (TLC) was performed as described by Hirmas et al. (2022) [46]. TLC was performed on F-60 silica plates (Merck, Germany) using 1-butanol/ethanol/ethanol/water 10:8:5 v/v as a run buffer and 1% orcinol in 10% H_2_SO_4_ in ethanol as the developer reagent. Two μL were taken from each sample. The chromatogram was developed after two runs and the sample was allowed to dry. The silica gel was heated at 100 °C until the bands were visually detectable. The carbohydrate consumption was evaluated in unidirectional (Lp, Bl1, Bl2, Bl3, and Bf supernatants) and bidirectional assays (“Lr vs. Lp,” “Bl1 vs. Lp,” and “Bf vs. Lp” supernatants).

### SCFA Quantification

SCFAs were quantified in selected supernatants from bidirectional and unidirectional assays at the end of the experiment (48 h) using a Lachrom liquid chromatograph (Merck-Hitachi) equipped with a UV detector at 210 nm. The Aminex HPX-87H ion exclusion column (300 mm, 7.8 mm; Bio-Rad) was operated with five mM H_2_SO_4_ at a flow rate of 0.45 mL/min at 35 °C for 35 min. Acetic, butyric, lactic, propionic, and succinic acid standards of known concentrations were used for column calibration (Sigma-Aldrich, St. Louis, MO, USA). Thirty microliters of the sample were injected and ran in duplicate. Data analysis was performed using Multi-HSM Manager software (Hitachi).

### Label-Free Comparative Proteomics

Pellets were obtained from bidirectional bacterial assays, and both monocultures and bicultures were analyzed (*n* = 4 biological replicates). Samples were lyophilized from 1.5 mL tubes in a 2.5 L lyophilizer at a temperature of − 50 °C (Labconco, USA) and stored at − 80 °C until further use. Extraction and proteomic analyses were performed following the methodology described by Caballero et al. (2022) [47]. Data were obtained from a Top15 method for MS/MS scans [48]. The label-free quantitative (LFQ) algorithm was used to normalize spectral intensities and calculate relative protein abundance [49], using MaxQuant software (v.1.6.15.9; https://www.maxquant.org/) [50]. Carbamidomethylation of cysteines was set as a fixed modification, whereas methionine oxidation and N-terminal acetylation were set as variable modifications. Maximum peptide/protein false discovery rates (FDR) were set at 1 % as the maximum compared to a reverse database. Perseus software (v.1.6.14.0) was applied for data organization and statistical analysis [51]. A *t*-test for quantitative analysis was used to compare the different batches with the control batch. Statistical differences were set at *p* < 0.05. A protein database of Lp, Lr, Bl1, and Bf from Uniprot (https://www.uniprot.org/) was used to perform the search. Qualitative analysis was performed by detecting proteins in at least two replicates from the same batch but none from the compared batch. ClueGO software [52] was used for gene ontology enrichment analysis [53]. To define term-term interrelationships and functional groups based on shared genes between terms, the Kappa score was set to 0.4. A minimum of three GO terms and 4% of covered genes were set to be selected. The *p* value was corrected using the Bonferroni downward step and was established as *p* ≤ 0.05 [52]. Fold change with respect to lactose was expressed as ΔlcLog_2_. When the protein was only found in one condition, the label-free quantitative (LFQ) intensity was expressed as Log_2._ Heatmaps containing those discriminant proteins with biological relevance were elaborated in R studio 4.2.2.

### Statistical Analysis

Multiple comparison ANOVA was performed for studies in SCFAs (2-factor ANOVA, Tukey’s test). In unidirectional assays, bacterial SCFAs in the supernatant were compared with the basal state, and SCFAs in bidirectional assays were compared biculture with monoculture. OD 600 of bidirectional assays (1-factor ANOVA) was obtained using GraphPad Prism 7 software. Statistical significance was set at *p* ≤ 0.05.

## Results

### Monoculture Assays and Enzyme Search

**Fig. 1:**
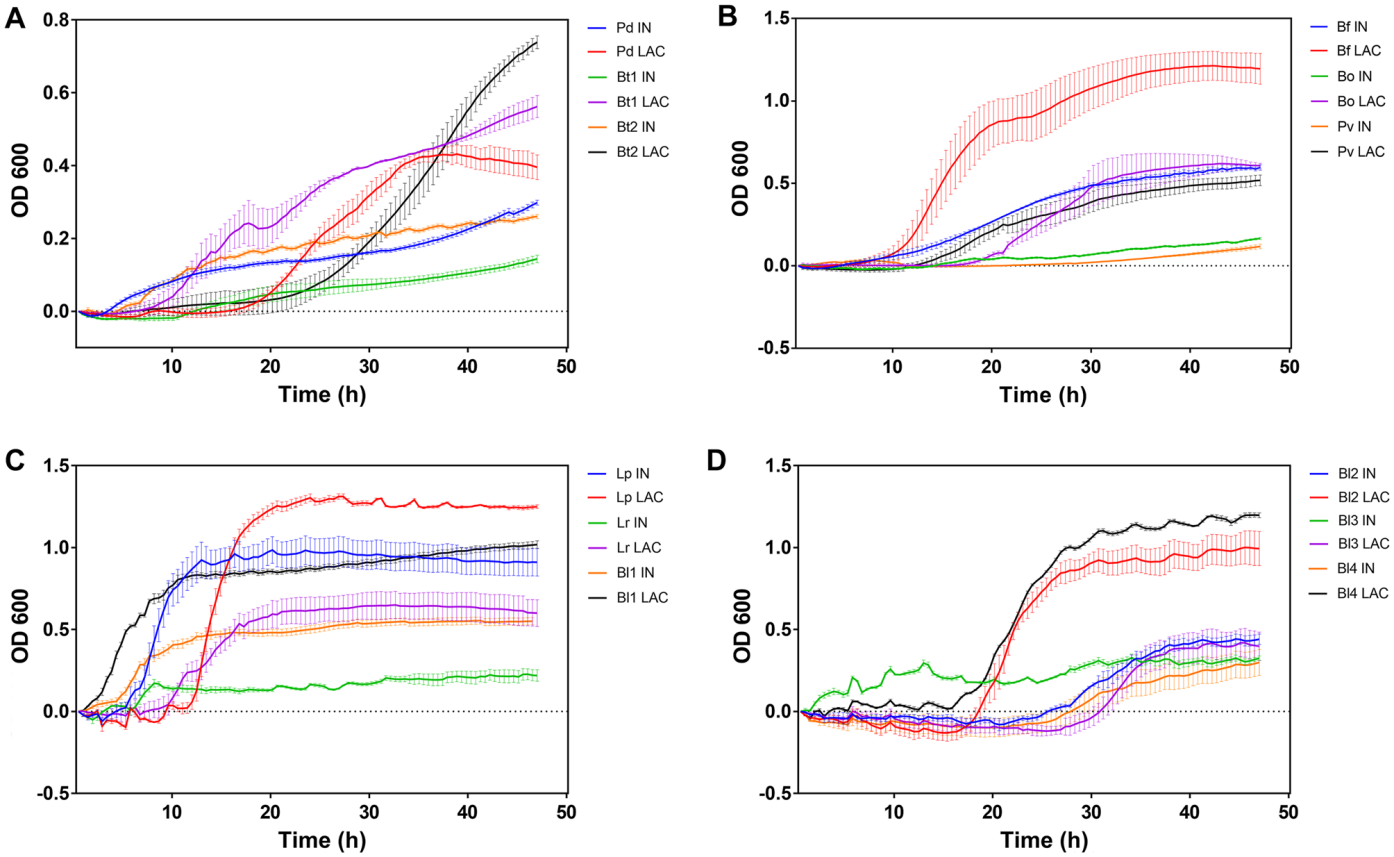
Bacterial growth curves of strains grown on inulin. IN: mZMB supplemented with inulin. LAC: mZMB supplemented with lactose. **A** The bacteria *Phocaeicola dorei* 5_1_36/D4 (Pd), *Bacteroides thetaiotaomicron* VPI-5482 (Bt1), and *B. thetaiotaomicron* HM23 (Bt2). **B** The bacteria *B. fragilis* HM714 (Bf), *Bacteroides ovatus* HM222 (Bo), and *Phocaeicola vulgatus* S1 (Pv). **C** The bacteria *L. paracasei* M38 (Lp), *L. ruminis* PT16 (Lr), and *B. longum* PT4 (Bl1). **D** The bacteria *B. longum* PT8 (Bl2), *B. longum* PT33 (Bl3), and *B. longum* PT7 (Bl4)

Figure 1 shows the growth of different gut microbes included in this work (Supplementary file Table S1), using inulin as the sole carbon source. All bacteria grew on this substrate with different vigorousness except *Bifidobacterium breve* I1 (Bb1), *Bifidobacterium bifidum* JCM-1254 (Bb2), *B. adolescentis* D3 (Ba), and *B. longum* SC664 (Bl5). The strains that grew best were Lp (ΔOD = 0.89), followed by Bf (ΔOD = 0.60), Bl1 (ΔOD = 0.55), Bl2 (ΔOD = 0.45), and Bl3 (ΔOD = 0.33). The results showed that inulin was widely consumed by different strains but differed in the degree of utilization according to their growth.

Table 1 shows the enzymes found in the bacteria that presented the highest growth on inulin (Lp, Bf, Bl1, and Bl2), which could be related to the fermentation of this dietary fiber. All the bacteria analyzed contained ABC transporters or PTS systems (necessary to transport the released sugars). Lp, Bl1, and Bl2 encoded sugar metabolism enzymes like sucrose-6-phosphate hydrolase and phosphofructokinase. Lp and Bf had a β-fructofuranosidase in their genome, an enzyme essential in inulin catabolism. The details of the enzymes found in the Lp genome were analyzed by Torres-Miranda et al. [21].

**Table 1:** Enzymes related to inulin metabolism found in the genomes of bacteria of interest, based on their growth on this substrate

**Bacteria**	**Enzymes**	**Reference**
*L. paracasei* M38	Sucrose-6-phosphate hydrolase, PTS system mannose/fructose, ABC transporter, 1-phosphofructokinase, glucose-6-phosphate isomerase, fructose 6-phosphate phosphoketolase, β-fructofuranosidase (3.2.1.26)	[27, 44, 45, 54]. And Uniprot.org was used to associate enzymes involved in inulin metabolism
*B. longum* PT4	Sucrose-6-phosphate hydrolase, PTS fructose transport; ABC transporter, fructose import, 6-phosphofructokinase, fructose-6-phosphate phosphoketolase	
*B. longum* PT8	Fructose-bisphosphate aldolase, PTS fructose transport; ABC transporter, fructose import, 6-phosphofructokinase, fructose-6-phosphate phosphoketolase	
*B. fragilis* 714	β-fructofuranosidase, PTS system sugar-specific permease component, ABC transporter, 6-phosphofructokinase, fructose-6-phosphate aldolase, fructose-1,6-bisphosphatase, fructose-bisphosphate aldolase pyruvate	

### Unidirectional Assays

The supernatants used as initial substrates were from the strains that grew best on inulin (Lp > Bf > Bl1 > Bl2 > Bl3). Table 2 summarizes the growth observed. Lp showed the highest growth in all spent supernatants (ΔOD of 0.99, 0.55, 0.76, 0.81 in Bl1, Bl2, Bl3, and Bf supernatants, respectively). On the other hand, the supernatant of Lp allowed little growth of the other bacteria. The bacteria that grew in the Bl1 supernatant were *Bacteroides* strains. In the supernatant of Bl2 and Bl3, this behavior was also observed. Finally, in the supernatant of Bf (Table 2), there was not much bacterial growth (except Lp).

**Table 2:** Unidirectional assay using Bacteria A supernatant after 24 h to feed Bacteria B. +: ΔOD between 0 and 0.09; ++: ΔOD between 0.1 and 0.19; +++: ΔOD between 0.2 and 0.44; ++++: ΔOD between 0.5 and 1; -: not growing; 0: not applicable. Other bacteria tested (Supplementary file Table S1) did not show any apparent growth

**Bacterium B**	**Inulin supernatant from**				
	**Bacterium A**				
	Lp	Bl1	Bl2	Bl3	Bf
Bo	-	+++	++	++	+
Bt1	+	+++	-	-	+
Bt2	+	+++	-	++	+
Bf	-	+++	+++	+++	0
Pv	-	++	+++	-	-
Pd	-	+++	+	+	-
Lp	0	++++	++++	++++	++++
Lr	-	++	-	+	+
Bl1	+	0	++	++	+
Bl3	+	+	-	0	+

**Fig. 2:**
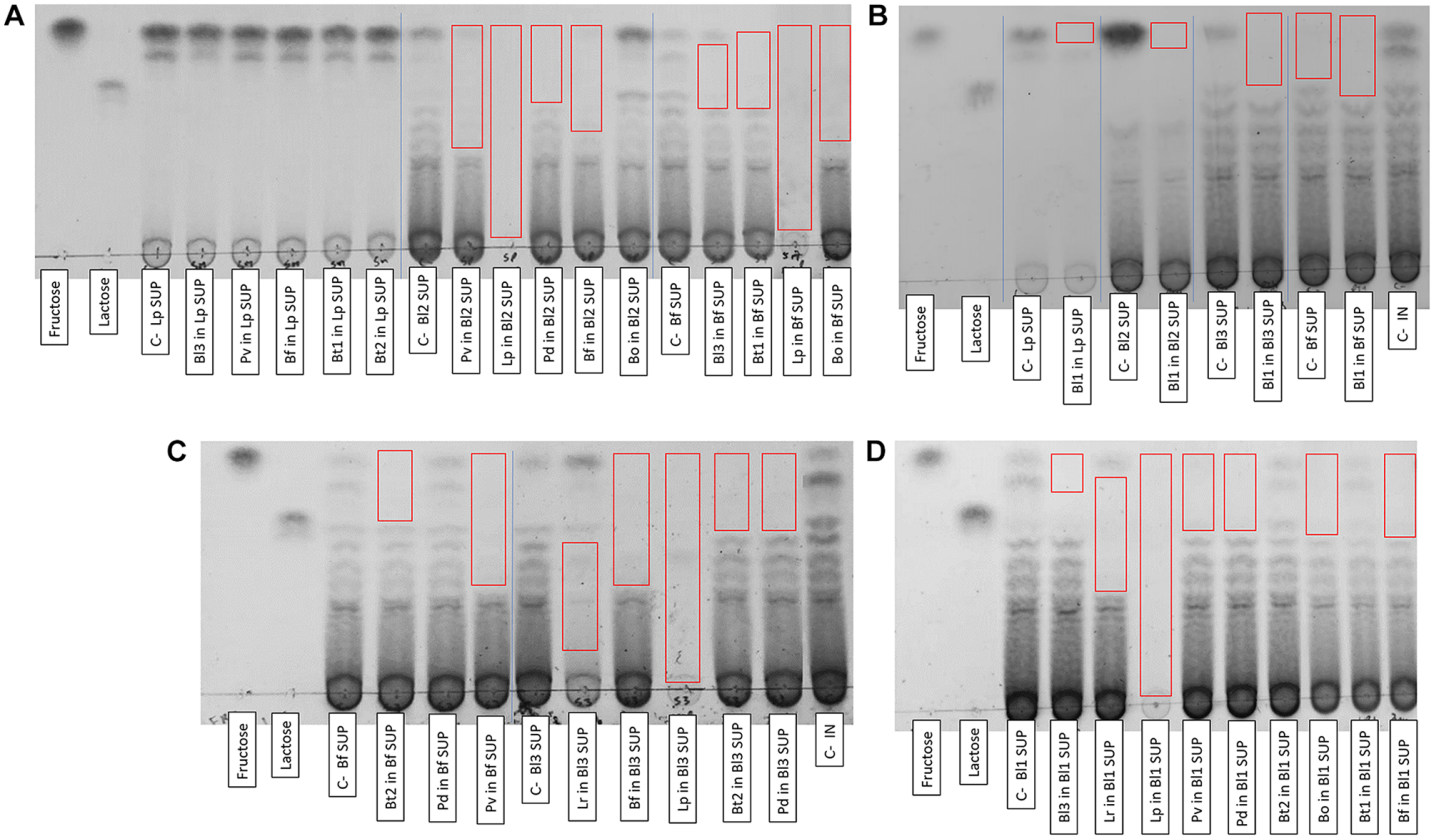
TLC of strains of interest using Lp (**A**, **B**), Bl1 (**D**), Bl2 (**A**, **B**), Bl3 (**B**, **C**), Bf (**A**, **B**, **C**) supernatants previously grown for 24 h on mZMB inulin 1%. SUP: Supernatant. C-: Initial supernatant (negative control). C- IN: mZMB inulin without bacterial growth. The red box indicates the fractions of inulin consumed

The TLC results of supernatants from unidirectional assays (Fig. 2) showed consumption mainly of monosaccharides or oligosaccharides derived from inulin. However, Lp was an exception, as it always consumed all fractions of inulin in the supernatants evaluated (Bl1, Bl2, Bl3, and Bf supernatants). In the supernatant of the Lp (Fig. 2A) other bacteria had no consumption, except for Bl1 (Fig. 2B), which consumed fructose. In the other supernatants, inulin metabolization preferences and partial degradation by *Bacteroides* and *Bifidobacterium* were observed in relation to oligofructose size. Specifically, the evaluated strains partially consumed small oligosaccharides in the Bl2 supernatant (Fig. 2A, B) and Bf supernatant (Fig. 2A–C). In the supernatant of Bl3, Bf consumed approximately half of the inulin (Fig. 2C), whereas Bl1 only consumed fructose (Fig. 2B). Fructose consumption was mainly observed in the Bl1 supernatant (Fig. 2D). On the other hand, among the SCFAs measured in unidirectional assays, acetate and lactate production predominated (Supplementary file Fig. S1).

## Bidirectional Assays

**Fig. 3:**
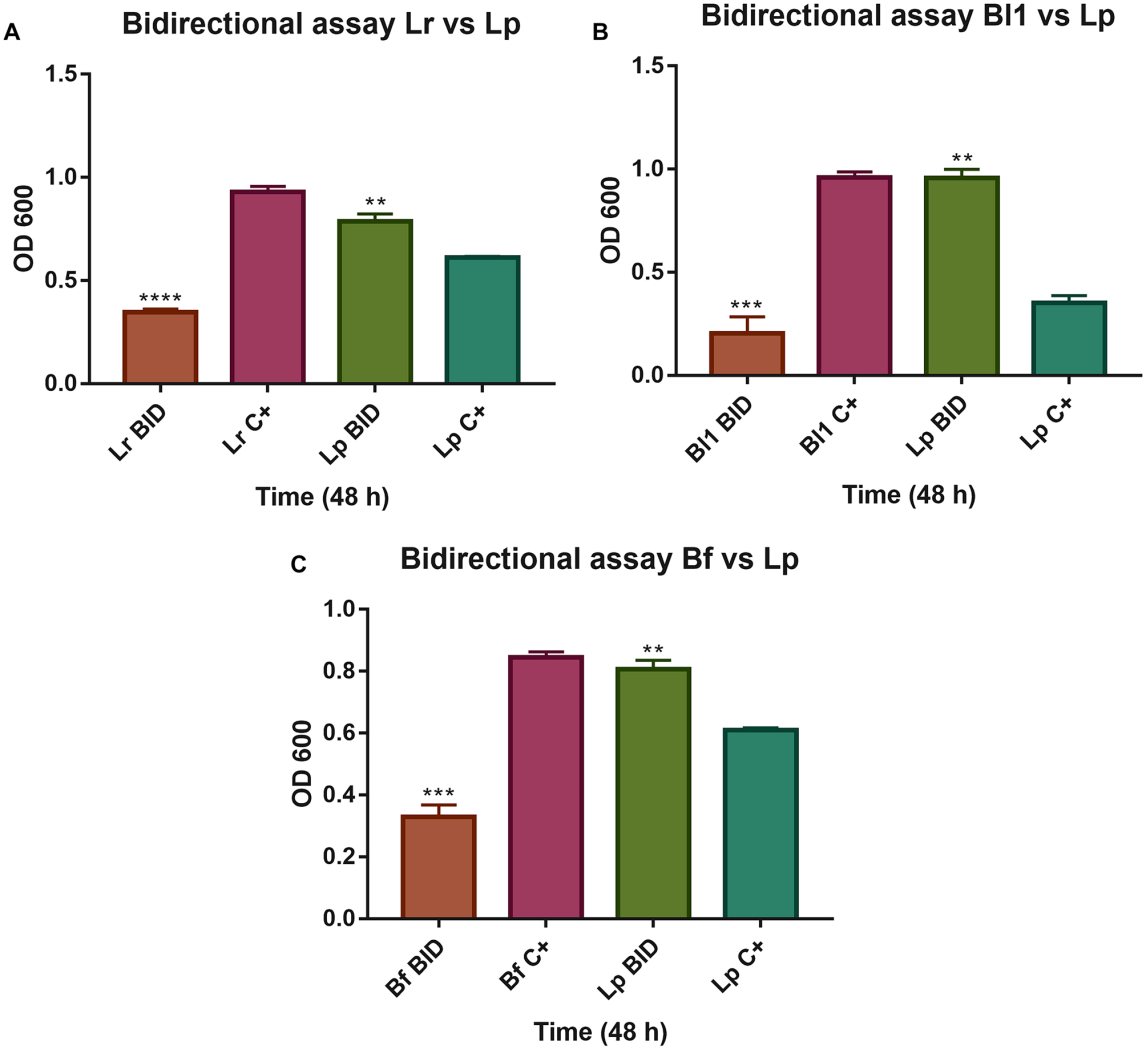
Bidirectional bacterial growth on inulin after 48 h. **A** Bidirectional assay of Lr and Lp. **B** Bidirectional assay of Bl1 and Lp. **C** Bidirectional assay of Bf and Lp. BID: Strain grown in a bidirectional assay, C+: Strain grew in monoculture. Lr: *L. ruminis* PT16. Bl1: *B. longum* PT4. Bf: *B. fragilis* HM714. Lp: *L. paracasei* M38. The monoculture was compared with the biculture in each strain. ANOVA statistical analysis was performed. **p < 0.01, ***p < 0.001, ****p < 0.0001

Later, we evaluated how a bacterium with high inulin consumption capacity (Lp) can impact the growth of other bacteria of different genera (*Lactobacillus*, *Bifidobacterium*, and *Bacteroides*) that also degraded inulin. Three bidirectional interactions were analyzed (Lr/Lp; Bl1/Lp; Bf/Lp). In the “Lr vs. Lp” growth (Fig. 3A), Lr decreased by 64%, and Lp increased by 30%, both in bicultures. A similar trend was observed in both “Bl1 vs. Lp” (Fig. 3B) and “Bf vs. Lp” (Fig. 3C) when grown in bicultures (with respect to monoculture), where Bl1 and Bf decreased (reduced by 79% and 61%, respectively), and Lp increased its growth (174%, and 31%, respectively). These results indicate that Lp resource competition dominated the three bacterial interactions evaluated.

**Fig. 4:**
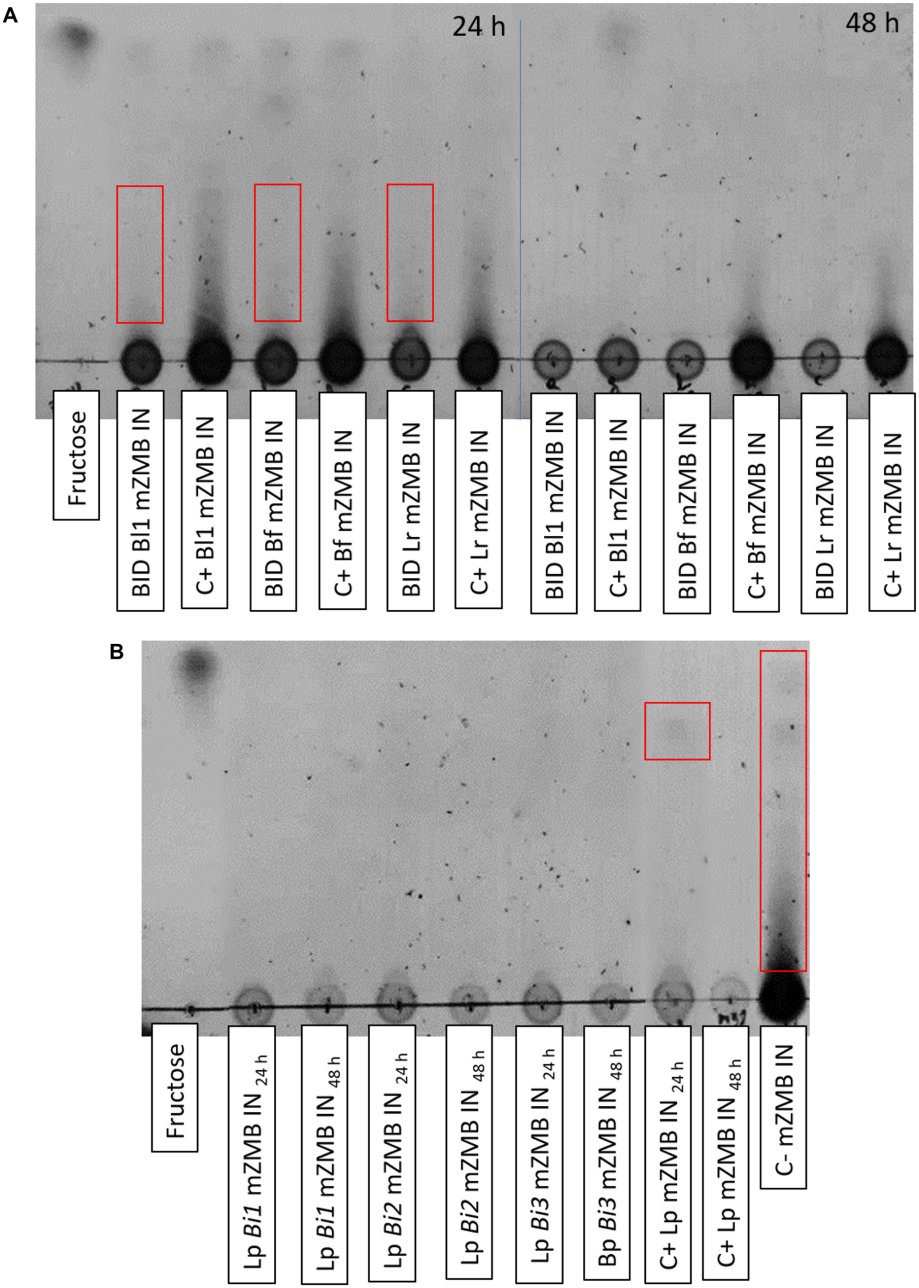
TLC of bidirectional assays of interest. **A** TLC of insert supernatants of Lr, Bl1, and Bf at 24–48 h. **B** TLC of well supernatants of Lp at 24–48 h. C+: Monoculture assay. BID, bidirectional assay; C- mZMB IN, mZMB inulin without bacterial growth; *Bi1*, biculture, in the presence of Lr; *Bi2*, biculture, in the presence of Bl1; *Bi3*, biculture, in the presence of Bf; Lr, *L. ruminis* PT16; Bl1, *B. longum* PT4; Bf, *B. fragilis* HM714; Lp, *L. paracasei* M38

Figure 4A shows the inulin consumption in the three metabolic interactions evaluated. Lr, Bl1, and Bf showed the same trend (Fig. 4A), where monocultures partially metabolized inulin at 24 h, and degradation was almost complete at 48 h. However, in the presence of Lp, inulin was almost completely utilized at 24 h because there was only an inulin remnant at the base of the TLC. And at 48 h, the inulin consumption was complete. As for Lp (Fig. 4B), only one oligosaccharide stayed at 24 h in the monoculture. At 48 h, the total substrate used was. In the bicultures of Lp with different bacteria, the total catabolism of inulin was observed (both at 24 h and 48 h).

**Fig. 5:**
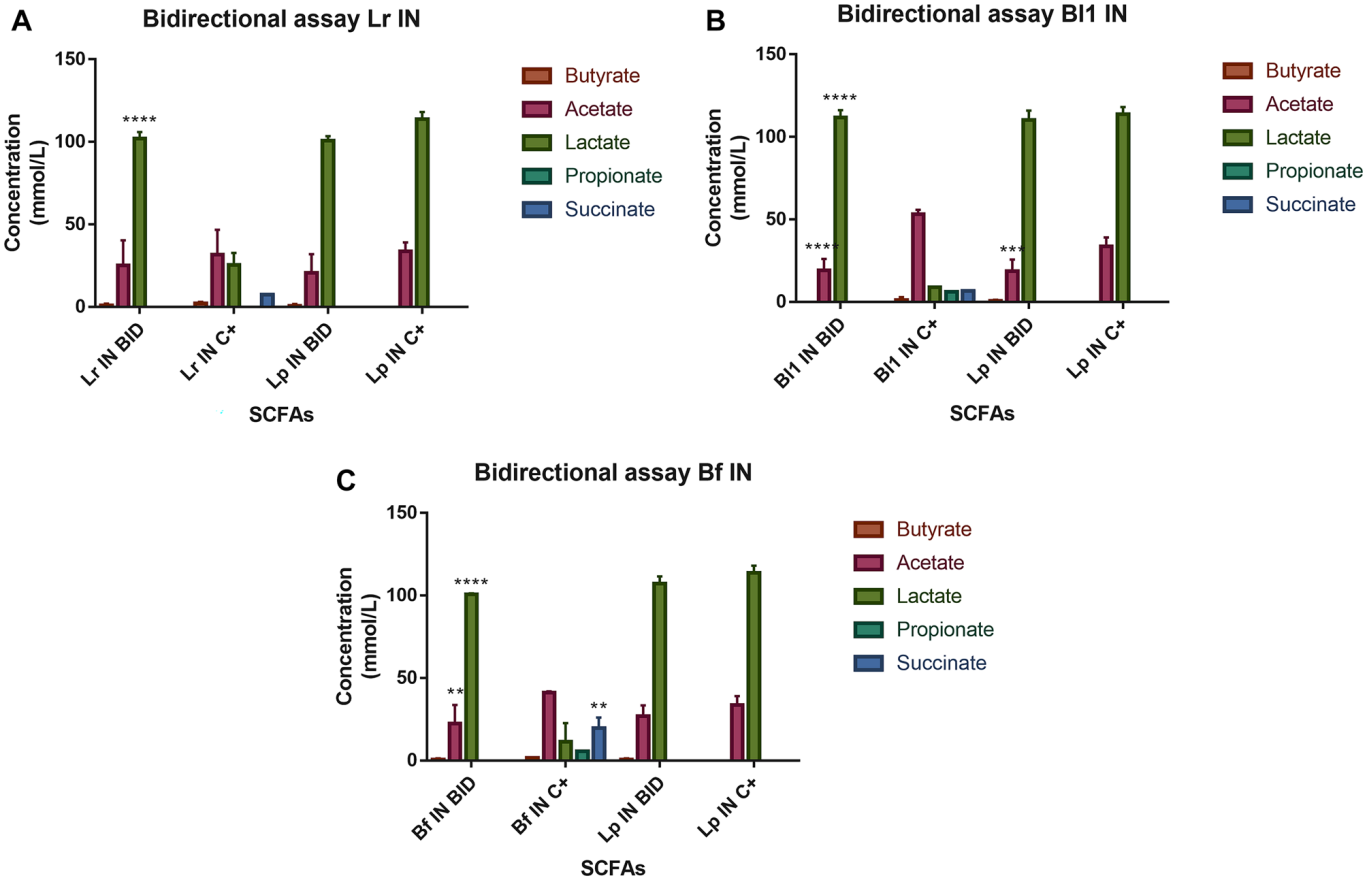
Quantification of short-chain fatty acids in bidirectional assays. ANOVA statistical analysis was performed. **p < 0.01, ***p < 0.001, ****p < 0.0001. IN, mZMB supplemented with inulin; C+, monoculture assay; BID, bidirectional assay; *Bi1*, biculture, in the presence of Lr; *Bi2*, biculture, in the presence of Bl1; *Bi3*, biculture, in the presence of Bf; *Bi4*, biculture, in the presence of Lp; Lr, *L. ruminis* PT16; Bl1, *B. longum* PT4; Bf, *B. fragilis* HM714; Lp, *L. paracasei* M38

Figure 5 shows the SCFAs produced in bidirectional assays, where the SCFAs were compared with their monocultures. These results indicated the absence of butyrate. Propionate and succinate were mainly detected when Bf was grown in a monoculture (Fig. 5C) but not in biculture. On the other hand, less acetate was detected in Lp supernatants in the presence of the three bacteria evaluated, since in monoculture it produced 33.7 mmol/L, and in biculture, it produced 20.6 mmol/L (in presence of Lr), 18.5 mmol/L (in presence of Bl1), and 26.9 mmol/L (in presence of Bf). In addition, acetate also declined in supernatants of other bacteria in bicultures, specifically decreased in Lr 20.5% (Fig. 5A), in Bl1 64.2% (Fig. 5B), and in Bf 45.4% (Fig. 5C). Finally, the amount of lactate remained little changed in the supernatant of Lp, but its concentration increased in the bicultures of other bacteria evaluated (Lr, Bl1, and Bf). However, this was probably due to the diffusion of this SCFA through the membrane, which corresponds to the lactate produced in high amounts by Lp.

## Label-Free Comparative Proteomics in Inulin

Proteomics used in this study was based on comparing cultures on mZMB IN with the basal state (mZMB LAC). Table 3 shows the four bacteria that were evaluated under different conditions. *L. paracasei* M38 (monoculture and bicultures in bidirectional assays, in the presence of Lr, Bl1, and Bf). In addition, proteomes from monocultures and bicultures in bidirectional assays (in the presence of Lp) were analyzed in *L. ruminis* PT16, *B. longum* PT4, and *B. fragilis* HM714 strains.

**Table 3:** Nomenclature of proteomics assays performed. P, *L. paracasei* M38; R, *L. ruminis* PT16; L, *B. longum* PT4; F, *B. fragilis* HM714; I, inulin; L1, lactose

**Bacterium 1**	**Bacterium 2**	**Substrate**	**Assay**
P	-	L1	PL1
P	-	I	PI
P	R	I	PRI
P	L	I	PLI
P	F	I	PFI
R	-	L1	RL1
R	-	I	RI
R	P	I	RPI
L	-	L1	LL1
L	-	I	LI
L	P	I	LPI
F	-	L1	FL1
F	-	I	FI
F	P	I	FPI

The proteomic assay showed that the diversity of metabolic pathways was higher in Lp monoculture (Supplementary file Fig. S2A) compared with bicultures. Specifically, this could be related to the increase in its OD shown in previous experiments. In the presence of Bf (Supplementary file Fig. S2D), Lp increased pathways suggesting an accelerated sugar consumption (carbohydrate derivate metabolic process and carbohydrate transport). This correlates with the increment in OD in co-culture. In general, bacteria competing with Lp decreased the number of metabolic pathways (Supplementary file Fig. S2). Carbohydrate metabolism from bacteria competing with Lp was negatively affected, which correlates with the low growth observed for these bacteria (Lr, Bl1, and Bf) in previous experiments (Fig. 3).

**Fig. 6:**
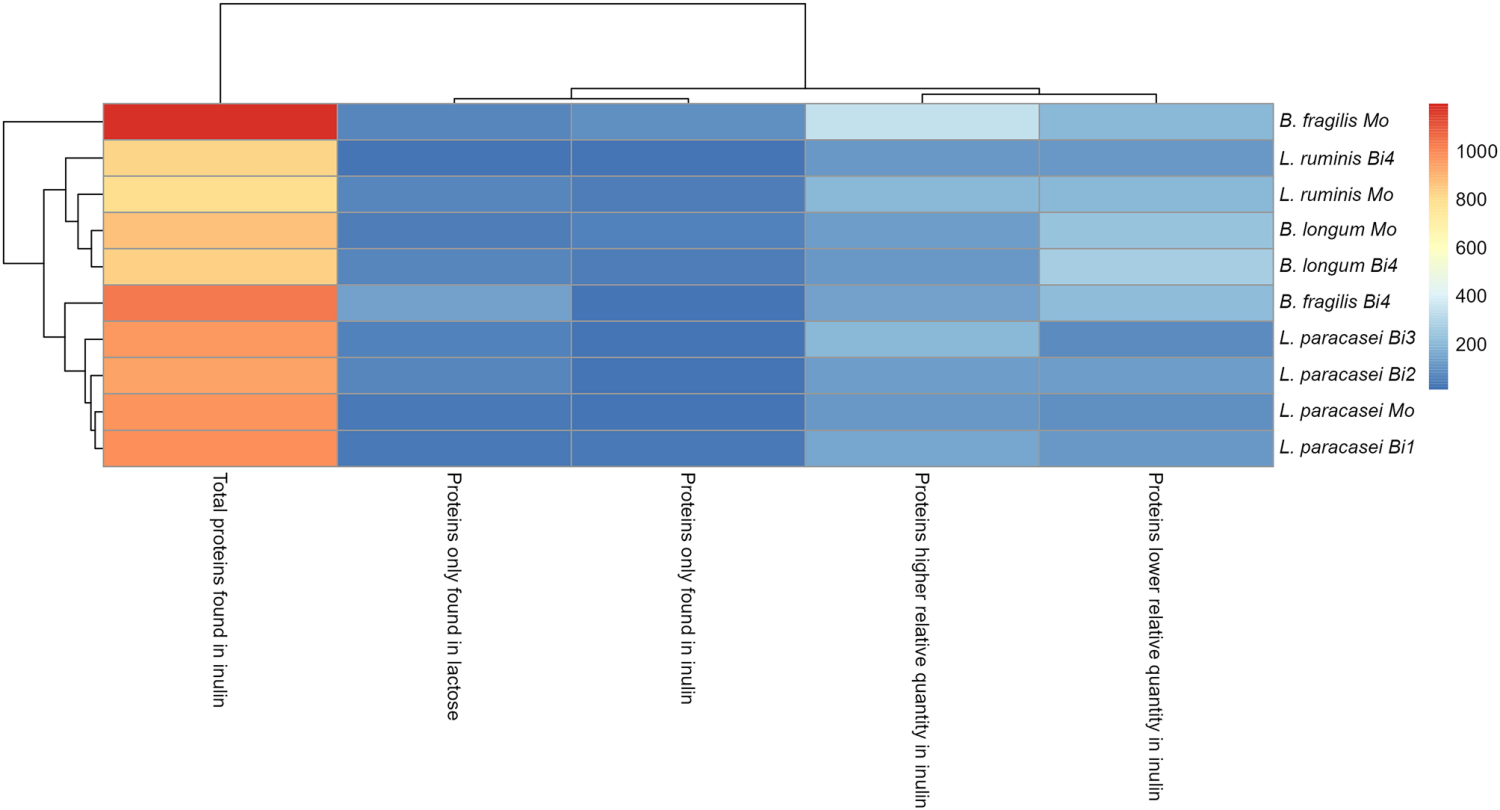
Heat map (hieratical clustering) based in the number of proteins in different conditions found in bacterial interactions when compared with the basal treatment, lactose. The *x*-axis labels indicate those conditions. For proteins found in higher or lower abundance, *p* value < 0.05. For proteins only found in one treatment, they were found in at least two biological replicates and not found in any of the replicates of the counterpart. *Mo*, monoculture; *Bi1*, biculture, in the presence of *L. ruminis* PT16; *Bi2*, biculture, in the presence of *B. longum* PT4; *Bi3*, biculture, in the presence of *B. fragilis* HM714; *Bi4*, biculture, in the presence of *L. paracasei* M38

Figure 6 shows the number of identified proteins that increased or decreased in quantity when comparing proteomes of bacteria grown in inulin to growth in lactose. Lp in biculture displayed a slight increase of total proteins in inulin (147 in the presence of Lr, 124 in the presence of Bl1, and 193 in the presence of Bf), with respect to monoculture (117). The greatest increment in these proteins was 65% when Lp grew in the presence of Bf. In contrast, bacterial strains in the presence of Lp decreased the proteins higher relative quantity in inulin concerning monoculture. Specifically, biculture proteins from Lr (106), Bl1 (117), or Bf (139), with respect to their monocultures (200, 119, 332, respectively). Bf was the bacterium that reduced most of the total protein in inulin (13%) in bicultures.

### Proteins Found in *L. paracasei* M38, When Grown on Inulin 

**Fig. 7:**
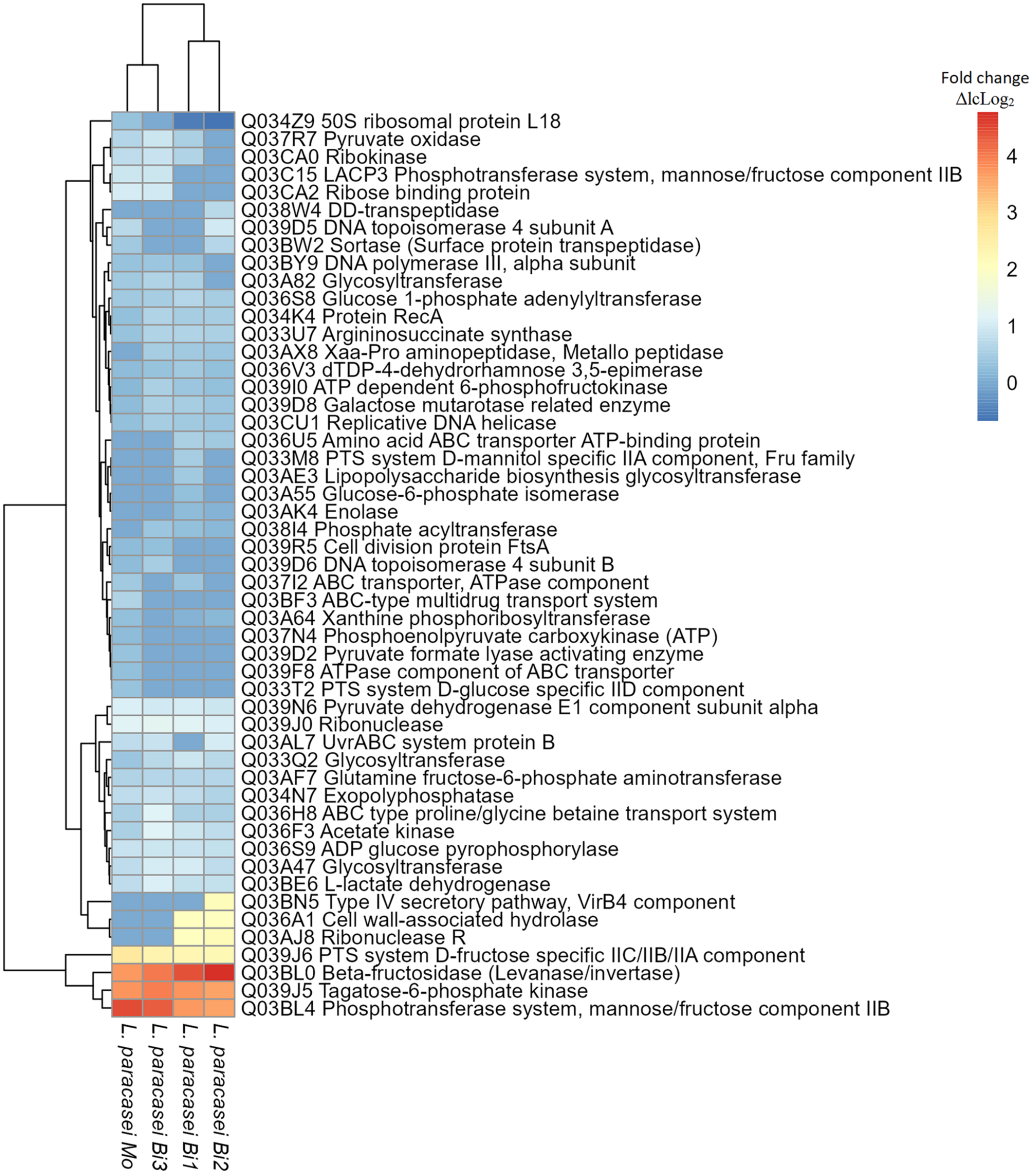
Heat map of proteins increased in abundance when compared with the basal medium, lactose (ΔlcLog_2_) in *L. paracasei* M38 in the four conditions analyzed. For proteins only found in one treatment, they were found in at least two biological replicates and not found in any of the replicates of the counterpart. *Mo*, monoculture; *Bi1*, biculture, in presence of *L. ruminis* PT16; *Bi2*, biculture, in presence of *B. longum* PT4; *Bi3*, biculture, in presence of *B. fragilis* HM714

In the three bicultures, Lp increased the proteins found in higher quantities when inulin was consumed, with respect to the monoculture (Fig. 7). Generally, these proteins were mainly related to sugar metabolism, such as glycosyltransferases or ABC transporters that can transport FOS into the cellular interior, and 6-phosphofructokinase (ΔlcLog_2_ 0.19 in PI, ΔlcLog_2_ 0.40 in PRI, ΔlcLog_2_ 0.30 in PLI, and ΔlcLog_2_ 0.57 in PFI), which participates in the phosphorylation processes of inulin degradation intermediates. A phosphotransferase system for fructose (ΔlcLog_2_ 4.46 in PI, ΔlcLog_2_ 3.70 in PRI, ΔlcLog_2_ 3.62 in PLI, and ΔlcLog_2_ 4.33 in PFI) and β-fructosidase/levanase/invertase (ΔlcLog_2_ 3.72 in PI, ΔlcLog_2_ 4.41 in PRI, ΔlcLog_2_ 4.77 in PLI, and ΔlcLog_2_ 4.02 in PFI) were found. They belong to the operon inulin-degrading *fosRABCDXE*, and β-fructosidase was higher in biculture than in monoculture, mainly increasing in the presence of Bl1, probably due to its strong competition for the substrate. In addition, 50S ribosomal protein L18 increased in the presence of Bl1, indicating accelerated growth. Proteins involved in cell proliferation, such as ribonuclease and cell wall-associated hydrolase, or conjugation proteins, such as the type IV secretion system (T4SS), were found only in some bicultures (Fig. 7). Other proteins found in greater numbers in Lp in bicultures were related to bacterial growth, such as DNA helicase and DNA polymerase III, which that are essential for DNA replication. In addition, were found protein RecA, which has DNA repair functions, DNA topoisomerase 4 that relaxes supercoiled DNA before replication, and cell division protein FtsA. Furthermore, in bicultures increased the acetate kinase (related to acetate production) and sortase (a surface protein). Sortase only increased its relative quantity in the presence of Bl1 (ΔlcLog_2_ 0.45 in PI, ΔlcLog_2_ 0.66 in PLI). Ribonuclease R, which is involved in RNA metabolism, increased in Lp in the presence of Lr and Bl1. Finally, enolase was only found in Lp when grown with Lr and Bl1. Among the proteins only found in Lp when grew on inulin (not found on lactose), relaxase and T4SS were only found in bicultures (concerning monoculture), in the presence of Lr, or Bl1 (Supplementary file Fig. S3).

### Proteins Found in *L. ruminis* PT16, *B. longum* PT4, and *B. fragilis* HM714, When Grown on Inulin

**Fig. 8:**
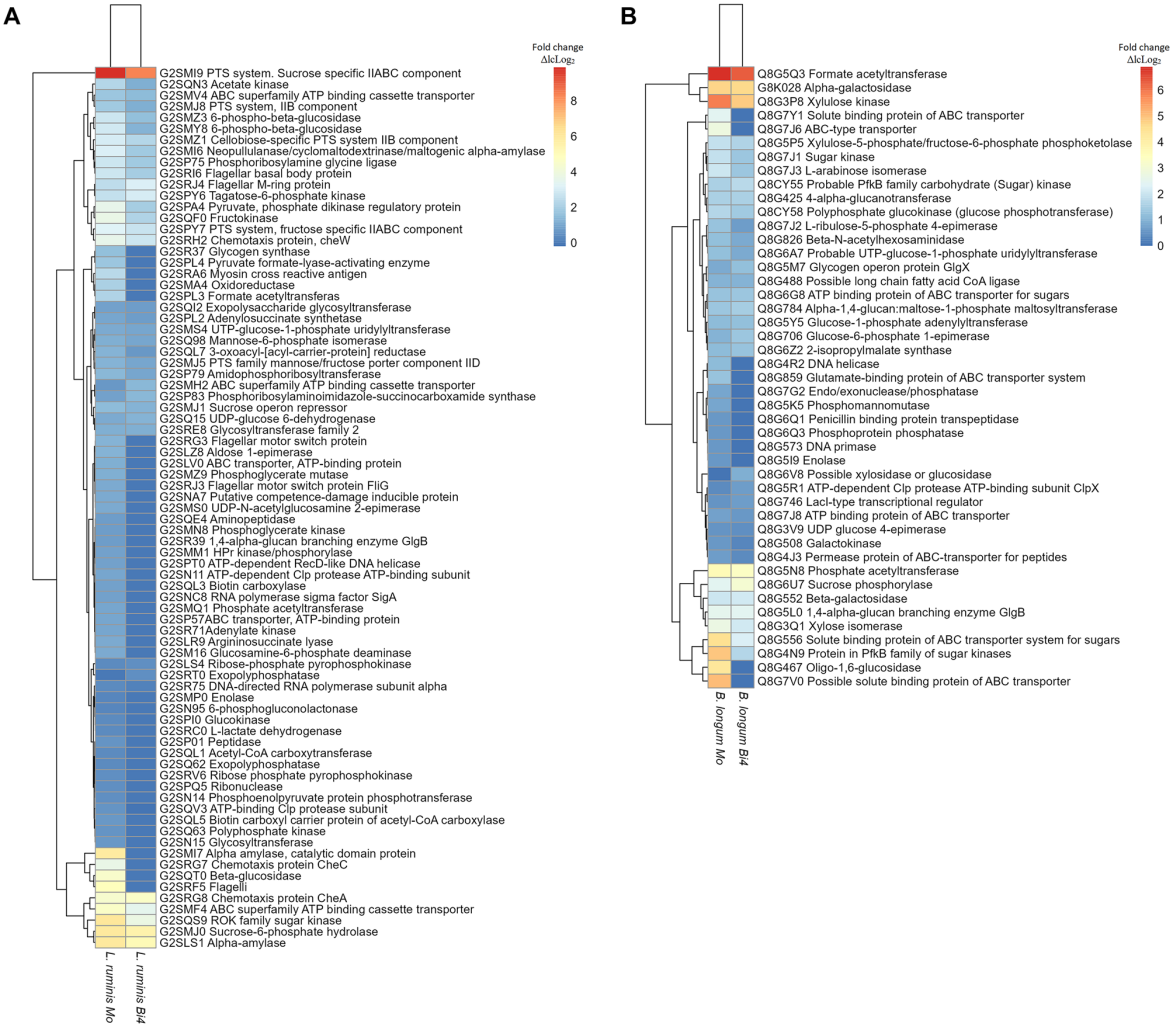

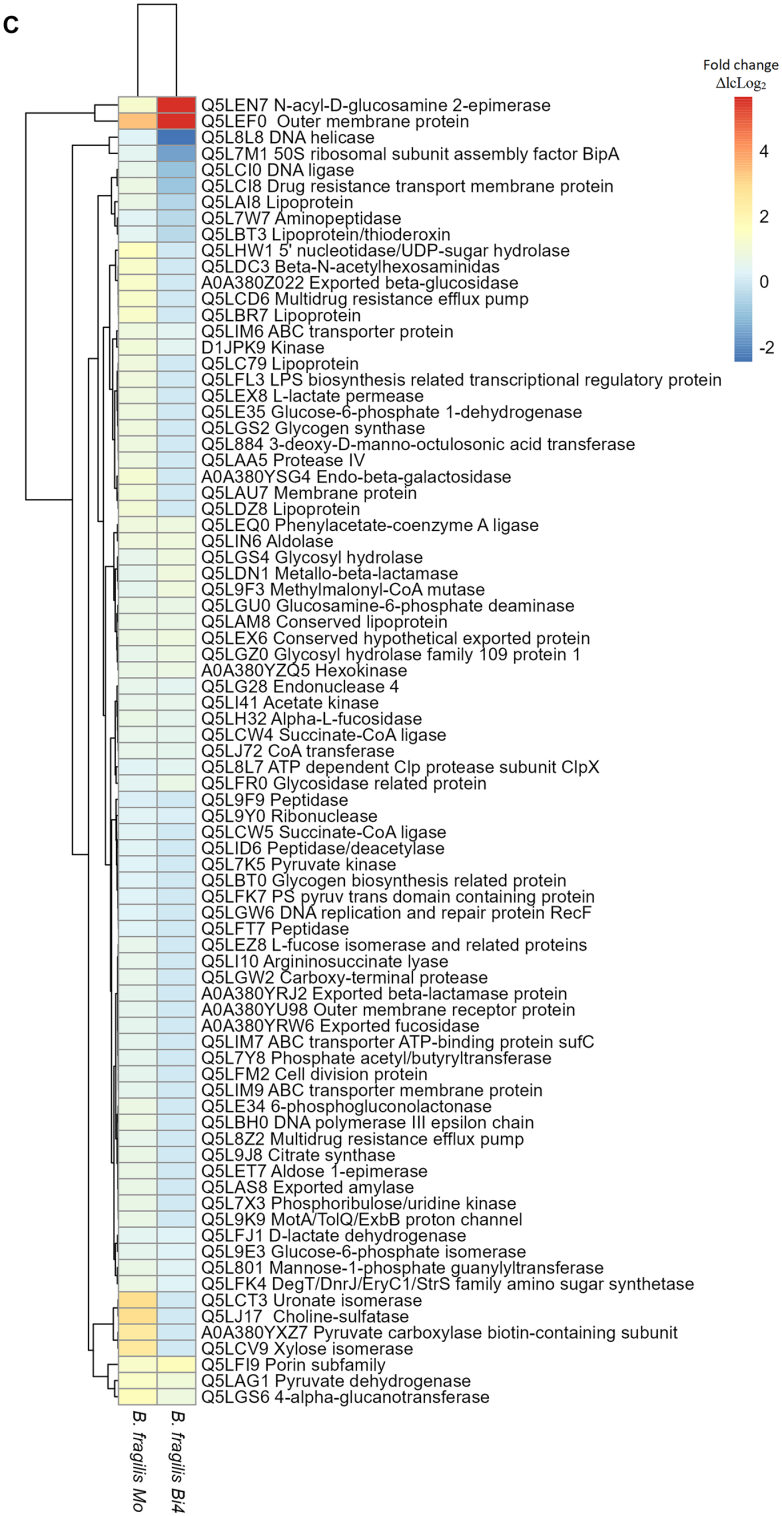
Heat map of proteins increased in abundance in *L. ruminis* PT16 (**A**), or *B. longum* PT4 (**B**), or *B. fragilis* HM714 (**C**) when compared with the basal medium, lactose (ΔlcLog_2_), in two conditions analyzed. For proteins only found in one treatment, they were found in at least two biological replicates and not found in any of the replicates of the counterpart. *Mo*, monoculture; *Bi4*, biculture, in presence of *L. paracasei* M38

In general, in the evaluated strains (Lr, Bl1, and Bf), the proteins of high relative quantity in inulin (Fig. 8) were reduced or not detected in the presence of Lp (bicultures) with respect to monoculture. Specifically, only in monocultures were found certain enzymes related to sugar consumption (not found in biculture probably due to competition with Lp). For example, ABC transporters (found in Lr, Bl1, and Bf), and proteins of glucose degradation such as β-glucosidase (found in Lr), oligo-1,6-glucosidase (found in Bl1), and exported β-glucosidase (found in Bf). Furthermore, proteins with several essential functions were found only in the monocultures. Some were helicase (found in Lr, Bl1, and Bf), repair protein RecF (found in Bf), DNA polymerase III (found in Lr), and DNA primase (found in Bl1) related to DNA replication. Flagellar proteins (found in Lr) were associated with bacterial movement, and succinate-CoA ligase (found in Bf) was related to succinate production.

On the other hand, among the proteins found in both monocultures and bicultures, a general decrease in fold change was observed in the presence of Lp. In Lr (Fig. 8A), among these proteins decreased in bicultures were of sugar transport (PTS family fructose, PTS system sucrose, and ABC transporter) or proteins of inulin degradation, such as fructokinase (ΔlcLog_2_ 3.67 in RI, ΔlcLog_2_ 2.21 in RPI), and sucrose-6-phosphate hydrolase (ΔlcLog_2_ 5.95 in RI, ΔlcLog_2_ 5.45 in RPI). In Bl1 (Fig. 8B), the proteins found were related to sugar transport, such as ABC transport and glucose phosphotransferase (both decreased in biculture). In addition, fructose-6-phosphate phosphoketolase (ΔlcLog_2_ 1.92 in LI, ΔlcLog_2_ 1.61 in LPI) was found, an enzyme relevant in bifid shunt metabolism [55]. In Bf (Fig. 8C), were found lipoproteins (decreased in biculture) and proteins that linked to DNA replication, such as DNA helicase (ΔlcLog_2_ 0.29 in FI, ΔlcLog_2_ -2.45 in FPI) and DNA ligase (ΔlcLog_2_ 0.62 in FI, ΔlcLog_2_ -0.95 in FPI).

Interestingly, in Bf, several enzymes related to sugar metabolism were found, but they were not directly involved in the degradation of inulin. Proteins involved in inulin degradation, such as levanase were found only in the monocultures (Supplementary file Fig. S4C). Finally, proteins were generally only found in bacteria when they grew on inulin (not found on lactose). More proteins were found in the monoculture than in the bicultures (Supplementary file Fig. S4). In summary, the effect of Lp, when interacting with the strains evaluated (Lr, Bl1, and Bf), manifested in the reduction of proteins relevant to the growth of the latter.

## Discussion

Plants rich in inulin lead to beneficial modifications in the composition and function of the intestinal microbiome [56]. Their effects on the human microbiome have been extensively studied, focusing on cooperative interactions with health-relevant bacteria. However, interactions between bacteria can be dominated by competition and depend on the substrate degradation capacity [57]. Single cultures showed vast consumption for the most part, except for four bifidobacteria (Bb1, Bb2, Ba, and Bl5). Although these bacteria did not grow on inulin, studies show that these species can consume fructans [58–60]. However, inulin size preference and consumption rate are strain specific [61, 62]. On the other hand, previous studies have shown that *Lactobacillus*, *Bifidobacterium*, and *Bacteroides* can grow on inulin [63, 64]. The growth of Lp was remarkable because it consumed inulin quickly and completely. This coincides with *L. paracasei* W20 [45].

In unidirectional assays (growth in supernatants), it was observed that Lp always consumed the substrate and showed the highest growth in the supernatants, repeating the behavior seen in monocultures. The TLCs (Fig. 2) supported these data because of the total sugar consumption of the supernatants by Lp. The other strains generally consumed the FOS remaining from the initial substrate (except for Lp, where Bl1 consumed fructose), which can be easily assimilated due to their small size. *B. longum* can use β-(2,1)-fructans, growing better with short-chain FOS than long-chain inulin [25]*.*

In bidirectional assays (Fig. 3), bacteria showed competition interaction in the three pairs evaluated (Lp with Lr, Bl1, or Bf), where Lp was always favored, probably due to the dominance of substrate consumption [65]. Although *L. paracasei* has been shown to have beneficial effects on other members of the intestinal microbiota [17, 64] and has been reported to allow growth on inulin of *Lactobacillus salivarius* W57 by cross-feeding. However, competing strains may be distantly related species or, conversely, differ only in a single mutation, depending on whether they overlap in resource use [34]. In addition, bacteria with similar nutritional requirements compete to acquire nutrients that are depleted in the environment [66]. The interaction most affected by competition was "Bl1 vs. Lp", where Bl1 reduced its growth more than the other strains, and Lp showed the opposite effect (174% increased with respect to monoculture). This may be due to the competition for fructose (observed in unidirectional TLC). The preference for this sugar has already been reported in the proteome of *B. longum* NCC2705 [67].

Interestingly, *Lactobacillus* is found in low amounts in the intestinal microbiome [68] but can alter the gut microbiome population [69]. Therefore, Lp reduced the growth of other strains. The highly competitive and nutrient-limited intestinal environment may be reflected in the consumption of inulin [70]. For example, *L. paracasei* populations reduced the survival of *L. monocytogenes, Salmonella enterica* subsp. *enterica*, and *E. coli* on inulin of artichokes foods [71]. The consumption of inulin was shown by TLC (Fig. 4) in Lr, Bl1, and Bf, where in bicultures, they showed accelerated degradation (compared to monoculture) due to Lp [45]. On the other hand, in bicultures, Lp (in the three conditions), Bl1, and Bf reduced acetate concentration (Fig. 5), which can be consumed as a carbon source [64] or decreased by low bacterial growth (of Bl1, or Bf). Furthermore, lactate increased in Lr, Bl1, and Bf due to the presence of Lp [72]. This change affects pH, an essential factor governing competition between bacterial species [73]. Finally, Bf in biculture did not produce succinate, prioritizing the use of the carbon source for primary metabolic pathways [74].

### Proteins Found in Lp

Bacteria in the presence of inulin increased the relative abundance of carbohydrate metabolism pathways [75]. In general, Lp increased the relative quantity of proteins found in inulin bicultures compared to monocultures (Fig. 7). As for proteins, Lp in biculture increased the ABC transporter, which is used in *Lactobacillus* to transport inulin or FOS to the cells [76, 77], and is degraded by cytoplasmic β-fructosidase [78]. In bicultures, increased 6-phosphofructokinase catalyzes the phosphorylation of D-fructose 6-phosphate to fructose 1,6-bisphosphate during inulin degradation [27]. In addition, proteins of the operon *fosRABCDXE* were found [21], phosphotransferase system fructose (PTS transport), and β-fructosidase/levanase/invertase (FosE), which hydrolyze the terminal non-reducing β-D-fructofuranoside residues in β-D-fructofuranosides.

In the presence of Bl1, Lp increased its β-fructosidase, which correlates with the best growth rates in previous trials (Fig. 3B). *L. paracasei* 1195 degrades FOS (DP < 10) extracellularly through β-fructosidase anchored in the cell wall. Each PTS transporter takes the released fructose and sucrose into the cells [79]. Proteomic analyses of* Lactobacillus plantarum* on inulin revealed an increase of β-fructosidase in monocultures [27], and *L. paracasei* consumed short-chain inulin using an exoinulinase enzyme (GH32) [45]. Furthermore, *L. paracasei* 1195 contains a cell wall-anchored β-fructosidase that degrades fructan outside the cell [27, 79].

As for proteins related to possible advantages in competition, was a sortase in PLI, which increased (regarding monoculture), functioning as an adhesin [80], increasing the chances of colonization [66]. Enolase was only found in Lp when grew with Lr and Bl1. This enzyme can also exhibit lyase activity [81]. Finally, the enzymes were found only in bicultures and not in lactose (Supplementary file Fig. S3), such as relaxase, binds to DNA and directs it to the recipient cells [82]. This can be complemented by T4SS (found only in the presence of Lr or Bl1). T4SS is used for genetic exchange in conjugation in *Lactobacillus* [83] and translocation of effectors with consequent impacts on genome plasticity [84, 85].

In summary, proteomic evidence showed that in bicultures, there was an increase in critical proteins for inulin degradation, bacterial growth (replicative DNA helicase and protein RecA), and phenotypic characteristics, which confer adaptive advantages to Lp when competing with other strains. These results suggest strong synergy between Lp and inulin. This performance has been shown in *L. paracasei* BGP1, together with inulin, contributing to the extension of the shelf life of foods, possibly due to competition or the production of antimicrobial compounds [24]. In addition, The symbiosis of *L. paracasei* I321 and inulin allowed a complete inhibition of *Salmonella* by antibacterial secretion and competitive adhesion [70].

### Proteins Found in Lr, Bl1, and Bf

In general, in all strains (Lr, Bl1, and Bf), certain proteins in the bicultures were not found or decreased with respect to those in the monocultures (Fig. 8). Because Lp had a higher prevalence of fermenting inulin [11]. ABC transporters and helicases were not detected in the presence of Lp. Only in the monoculture of Lr were flagellar proteins found that can provide motility in competition. This affects the ability of some bacteria to compete, whereas other bacteria use active locomotion to avoid competition [66]. Furthermore, succinate-CoA ligase was found only in FI and was correlated with succinate reduction in bicultures (Fig. 5C). This effect was contrary to cross-feeding, where the proteome of *B. fragilis* has been observed after consuming bifidobacterial EPS and activating the succinate pathway [86]. As for proteins in all bicultures, enzymes decreased related to inulin degradation or DNA replication, with respect to the monoculture (Fig. 8).

Interestingly, only in FPI was found transporter efflux component protein associated with antimicrobial resistance [87], probably because of Lp. In summary, all bacteria were negatively affected by Lp. However, the inhibitory effect on gram-positive bacteria (Lr, Bl1) was mainly based on reducing their ability to metabolize inulin. While in Bf, it primarily affected their growth, thereby affecting DNA replication. The inhibition of their growth by Lp drastically affected the production of many important enzymes, such as levanase (Supplementary file Fig. S4). Furthermore, it is known that *Bacteroides* spp. grows less when inulin is present at an acidic pH because it is an essential factor of competition between bacteria [73]. In this case, lactate was produced by Lp. Inulin regulates the gut microbiome composition and promotes the proliferation of beneficial bacteria [3]. But, when a competitive strain dominates the community, it extinguishes the weaker strain [66, 88].

## Conclusions

This work demonstrated how intestinal bacteria could modify their growth, proteomes, and sugar consumption when interacting. Unidirectional assays showed partial degradation of inulin by certain bacteria. It favors the coexistence of other microorganisms, which consume oligosaccharides. However, bidirectional assays showed that competition is preferred when a bacterium completely degrades the prebiotic substrate. In this context, *L. paracasei* M38, when interacting with different commensal bacteria (*L. ruminis* PT16, *B. longum* PT4, and *B. fragilis* HM714), it competed for the inulin (carbon source) and modified its proteome. The antagonistic effects favored *L. paracasei* M38, which increased the abundance of relevant enzymes in inulin catabolism, such as β-fructosidase, and sugar transporters. These proteins gave an adaptive advantage for inulin consumption over other bacteria evaluated. These latter reduced the number of proteins crucial for their development, leading to their poor growth. The synergy of inulin and *L. paracasei* M38 allows enhancing this bacterium to search for probiotic characteristics that displace the harmful host bacteria by competitive inhibition or other mechanisms.

### Supplementary Information

Below is the link to the electronic supplementary material.

Supplementary file1 (RAR 8499 KB)

## Data Availability

The datasets are available from the corresponding author on reasonable request.
